# Supporting Medication Adherence in Parkinson's Through Education: A Scoping Review of Interventions

**DOI:** 10.1111/hex.70852

**Published:** 2026-08-30

**Authors:** Hazel Haworth, Gillian Carter, Katherine M. A. Rogers, Gary Mitchell, Laura Jacobs, Patrick Stark

**Affiliations:** ^1^ School of Nursing and Midwifery Queen's University Belfast Belfast Northern Ireland UK; ^2^ Parkinson's UK London England UK

**Keywords:** behaviour change, educational interventions, medication adherence, Parkinson's disease, patient education, self‐management

## Abstract

**Background:**

Medication adherence in Parkinson's is complex, time‐sensitive, and influenced by clinical, behavioural, and contextual factors. Educational interventions have attempted to support medication adherence; however, the scope, focus, and effectiveness of such interventions remain unclear.

**Methods:**

This scoping review aimed to identify and map educational interventions for people with Parkinson's that included medication‐related education. Seven databases were searched with no date restrictions. Quantitative, qualitative, and mixed‐methods studies were eligible. Data were charted and synthesised using descriptive mapping and reflexive thematic analysis to explore intervention characteristics, reported evidence relating to medication adherence, intervention outcomes, and reported barriers and facilitators.

**Results:**

Eight papers reporting seven educational interventions were included. Interventions varied widely in content, delivery mode, and intensity, with medication education often embedded within broader programmes rather than delivered as a standalone intervention. Evidence regarding the potential of educational interventions to support medication adherence was reported, although this was based on a limited number of studies using direct adherence measures and more commonly on proxy indicators such as changes in knowledge, beliefs, and self‐management behaviours. Outcome measures were heterogeneous, limiting comparability. Improvements were more commonly observed in understanding, confidence, and communication than in sustained symptom outcomes. No study reported explicit use of behaviour change theory or co‐design approaches involving people with Parkinson's or their informal carers.

**Conclusions:**

Educational interventions show potential to support Parkinson's medication adherence, particularly through mechanisms conceptually aligned with self‐efficacy. Future interventions may be strengthened by explicit use of behaviour change theory and/or meaningful co‐design with people with lived experience.

**Lived Experience or Public Contribution:**

A Research Involvement Manager from Parkinson's UK is a co‐author and contributed to the design, conduct, and interpretation of this review, representing the perspectives of people living with Parkinson's. An advisory group comprising people with Parkinson's, carers, and healthcare professionals informed the wider research programme and reviewed the findings of this study. While no substantive changes were requested, this process supported interpretation and relevance to lived experience. Consistent with these perspectives, the review highlights a lack of co‐design in existing interventions and advocates for greater involvement of people with Parkinson's in future intervention development.

## 1 Introduction

Parkinson's is a rapidly growing neurological condition associated with substantial global disease burden [1]. Throughout this paper, the term Parkinson's is used in preference to Parkinson's Disease, reflecting terminology preferred by people living with the condition [2]. Parkinson's presents with a wide range of motor and non‐motor symptoms which can be improved with medication; however, medication regimens are often complex. Treatment is lifelong and subject to regular modifications as the condition progresses [3] and the medications become less effective [4], with unpleasant side effects becoming more prominent as dosages increase [5]. Co‐morbid conditions are also common [6], further increasing regimen complexity.

Parkinson's medications must be taken at specific times and dosages, as deviations can lead to worsening of symptoms [7]. National Institute for Health and Care Excellence (NICE) quality standards define timely administration of levodopa as within 30 min of the prescribed time [8]. Delays beyond this have been associated with deterioration in symptoms, with individuals reporting episodes of immobility, impaired communication, and swallowing difficulties [8, 9].

Although Parkinson's medication can ease symptoms, treatment does not necessarily restore full health or wellbeing [5]. As a result, people often organise their lives around medication routines, scheduling activities to coincide with periods of maximal therapeutic benefit [5], while also adapting medication use itself to fit their activities, for example by temporarily increasing a Produodopa pump dose for a large meal.

Complex regimens and the embodied burden of living with Parkinson's influence medication‐taking practices [5, 10]. Studies of intentional and unintentional non‐adherence identify a range of contributing factors such as forgetfulness [11], non‐motor symptom burden [12], regimen complexity and multiple daily dosing [13], and beliefs about medications [14]. Up to 70% of people with Parkinson's may not fully adhere to their prescribed medication schedules [13]. The World Health Organisation (WHO) defines adherence as the extent to which a person's behaviour corresponds with agreed recommendations from a healthcare provider [15]. In this paper, medication non‐adherence is therefore defined as any deviation from the prescribed Parkinson's medication regimen, including delayed, missed, or incorrectly timed doses, regardless of formulation or delivery method.

Previous reviews have examined factors associated with medication non‐adherence in Parkinson's [3], how information affects people living with the condition [16], and the effectiveness of self‐management interventions [17]. An early review of wider Parkinson's medication‐adherence interventions, reported as a conference abstract, identified promising findings across approaches spanning education, adherence therapy, a tracker app and pharmacist intervention [18]. However, these older reviews synthesised limited evidence on the nature of education relating to medication adherence. The scope and outcomes of educational interventions intended to support Parkinson's medication adherence remain unclear.

Non‐adherence is more common among people with Parkinson's and their informal carers who lack medication‐related knowledge [11, 19, 20]. Improving access to clear, timely information may therefore represent an important component of adherence support. The WHO recognises health education as a means of improving quality of life [21]. However, the nature, scope, and effectiveness of educational interventions addressing Parkinson's medication adherence have not been mapped or synthesised. Educational approaches vary widely in aim, delivery, and theoretical foundation, and medication education is often embedded within broader Parkinson's programmes rather than explicitly designed to support adherence. For this review, educational interventions were defined as structured approaches intended to increase understanding or support medication‐management knowledge, beliefs, skills, or behaviours among people with Parkinson's and/or their informal carers. These included therapeutic education, nurse‐led education and support, digitally delivered education, and medication‐related education embedded within broader self‐management, rehabilitation, or multidisciplinary programmes. Mapping this evidence base is necessary to inform the development of future educational interventions that reflect the complexity of Parkinson's medication management.

The aim of this mixed‐methods scoping review is to identify and describe educational interventions for people with Parkinson's that include medication‐related education, and to map their characteristics, reported outcomes, and barriers and facilitators relevant to medication adherence.

## 2 Methods

### 2.1 Study Design

A mixed‐methods scoping review was conducted to map the available evidence on educational interventions for people with Parkinson's that include medication‐related information. A scoping review approach was considered appropriate given the broad exploratory objectives, anticipated heterogeneity of interventions and outcomes, and the need to map the extent and nature of the available evidence rather than evaluate intervention effectiveness [22, 23]. The review aimed to map and describe the characteristics, reported outcomes, facilitators and barriers associated with educational interventions, and to identify gaps to inform future co‐design and intervention development. This scope was limited to peer‑reviewed studies of evaluated educational interventions; educational resources and supports available outside the research literature were therefore excluded.

This review followed the six‐stage methodological framework developed by Arksey and O'Malley [22] and subsequently refined by Levac et al., [23] and Colquhoun et al., [24]. Stages 1–5 were undertaken and reported here to map the nature, range, and extent of relevant research. Although a formal consultation stage was not conducted, findings informed subsequent quantitative, qualitative, and co‐design phases of the wider research programme. Reporting followed PRISMA‐ScR guidelines [25], with the checklist provided as Table S1. An a priori protocol was developed to guide the review but was not prospectively registered or published. As this scoping review used evidence available in the published literature, ethical approval was not required.

### 2.2 Research Questions

The overarching question to be answered by this scoping review was:

- What does previous research tell us about educational interventions to support people living with Parkinson's for medication adherence?

To address this question, the review examined the following sub‐questions:

- RQ1 What different types of educational interventions have been developed to support medication adherence for people with Parkinson's?
- RQ2 What evidence has been reported regarding the potential of educational interventions to support medication adherence in people living with Parkinson's?
- RQ3 In addition to medication adherence, what other outcomes have been used to assess the effectiveness of educational interventions about medication adherence for people living with Parkinson's?
- RQ4 What barriers and facilitators for the successful implementation of educational resources to support Parkinson's medication adherence can be identified?

### 2.3 Eligibility Criteria

Eligibility criteria were defined using the Population, Concept, and Context (PCC) framework [26] with Population defined as Parkinson's, Concept as medication adherence, and Context as education. Studies were eligible if they reported an educational intervention for people living with Parkinson's, and/or their informal carers, that included medication‐related education relevant to adherence.

Qualitative, quantitative, and mixed‐methods peer‐reviewed studies were eligible to capture a broad range of evidence, including intervention characteristics, reported outcomes, and barriers and facilitators. Interventions aimed at educating healthcare professionals were excluded as beyond the scope of this review. Studies were limited to those published in English, with no restrictions on publication date to enable comprehensive mapping of the available evidence; the earliest included study was published in 2001.

### 2.4 Search Strategy

An iterative search process was undertaken to identify relevant studies, consistent with scoping review guidance that supports refinement of search strategies as familiarity with the literature develops [22, 23]. The final search strategy was refined with input from a subject librarian at the research team's institution.

Searches were conducted between April and May 2024 across the following databases: CINAHL, PsycINFO, Cochrane Library, MEDLINE, Embase, Web of Science, and Scopus. To ensure currency, the search was repeated in January 2026; no additional eligible studies were identified. The full search strategy, including database‐specific search terms, is provided in the Table S2.

### 2.5 Study Selection

Search results were uploaded to Covidence systematic review software [27], where duplicates were automatically removed prior to screening.

**Figure 1:**
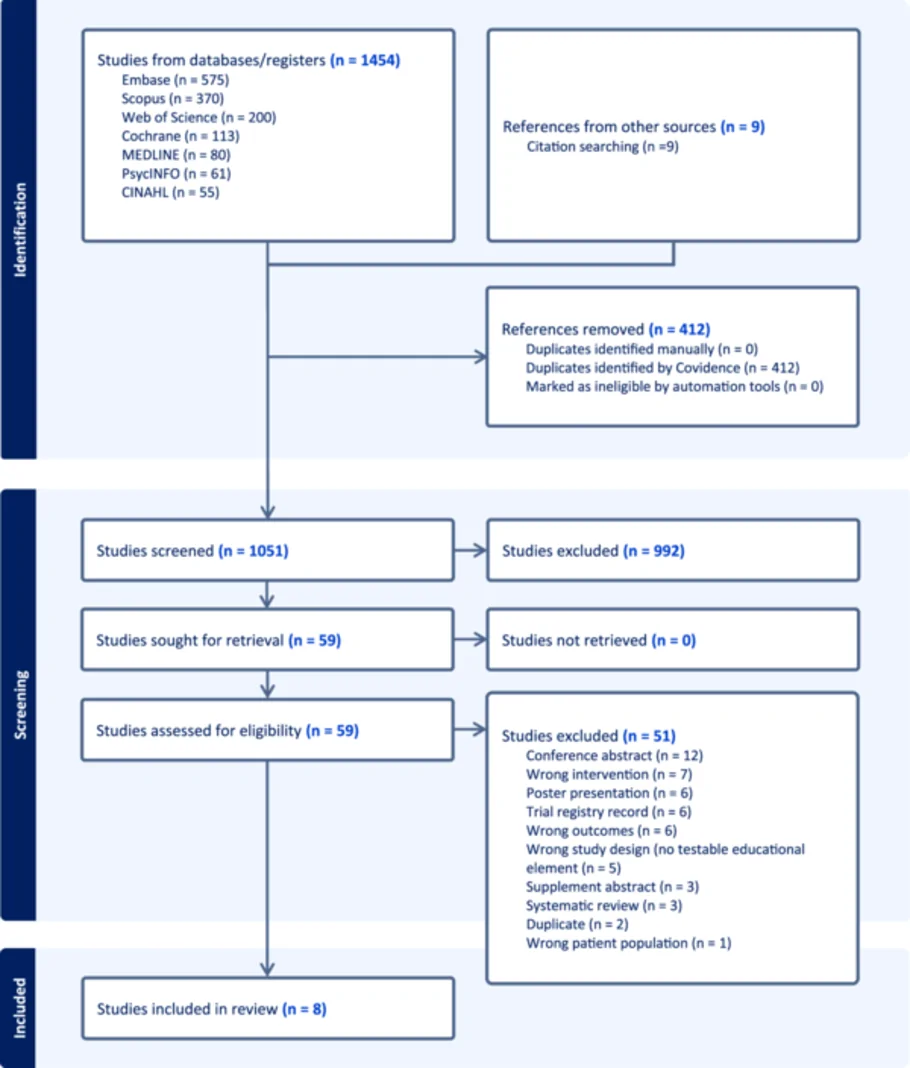
Flow diagram of study selection. Flow diagram illustrating the identification, screening, eligibility assessment, and inclusion of studies, reported in accordance with PRISMA‑ScR guidelines.

Study selection followed a two‐stage screening process. Title and abstract screening of 1,051 records was undertaken by one reviewer as part of a doctoral research programme. To support consistency, the search strategy and eligibility criteria were developed collaboratively by the research team, pilot searches were reviewed collectively, and the data‐charting framework was piloted and refined through team discussion. Fifty‐nine papers progressed to full‐text assessment against the predefined eligibility criteria. Full‐text eligibility decisions and the final set of included papers were reviewed by a second researcher, with uncertainties resolved through discussion. Following full‐text assessment, 51 papers were excluded. Review articles identified during full‐text screening were excluded but screened for relevant primary studies. Eight papers met the inclusion criteria and were included for data extraction. The study selection process is summarised in Figure 1.

### 2.6 Data Charting

Data charting was conducted using a structured extraction framework developed to capture key characteristics of the included studies, consistent with scoping review methodology [22, 23]. Data were extracted using Covidence and included general study information (e.g., author, year, country), study characteristics (setting, participants, design, and intervention description), and reported outcomes.

The extraction framework was piloted on three studies and refined through team discussion to ensure alignment with the review questions. Additional fields were added to capture intervention delivery and implementation, including mode of delivery, sample size, reported facilitators and barriers, study limitations, and recommendations for future research or practice. This iterative process supported comprehensive and consistent charting of data across studies [22, 23].

### 2.7 Data Synthesis

Data synthesis was tailored to each research question, using descriptive mapping and thematic approaches as appropriate, in line with scoping review guidance [22, 23]. Descriptive mapping was used to address Research Questions 1 and 3, which focused on the types of educational interventions developed and the outcomes used to assess them. These findings were summarised using qualitative content analysis.

Research Question 2, which explored evidence regarding the potential of educational interventions to support medication adherence, was analysed using reflexive thematic analysis to identify patterns in how adherence‐related findings were reported across studies. Research Question 4, which focused on barriers and facilitators to the successful implementation of educational resources, was also analysed using reflexive thematic analysis.

Thematic analysis was conducted inductively, with themes developed from the charted data and refined through an iterative, reflexive process. This approach enabled a more interpretive exploration of patterns across studies, complementing descriptive mapping undertaken for other review questions [28, 29].

### 2.8 Patient and Public Involvement and Engagement

Patient and public involvement (PPI) was embedded throughout this research. A co‐author is the Research Involvement Manager of a major charity which supports and advocates for people living with Parkinson's and their carers (Parkinson's UK). This has ensured the conception, design, and interpretation of this study reflect the priorities and perspectives of people living with Parkinson's and those who care for them.

An advisory group comprising people with Parkinson's, carers, and Parkinson's professionals were active contributors to the study of which this review forms a part. This group informed the overall direction of the research and provided input to each study phase.

Following completion of the review, findings were shared with members of the advisory group to support interpretation and ensure relevance to lived experience. This provided an opportunity for feedback on the findings and their implications.

The perspectives of people living with Parkinson's also informed the interpretation of gaps in the literature. In particular, the absence of co‐design in existing interventions aligns with advisory group member views and underpins the recommendation that future interventions should be developed in partnership with people with lived experience.

## 3 Results

Eight papers met the inclusion criteria, representing seven educational interventions designed to support medication‐related education for people with Parkinson's. The studies were published between 2001 and 2023 and conducted across the UK, USA, France, Sweden, and Italy. Interventions varied in aim, delivery, and theoretical underpinnings, with medication education often embedded within broader Parkinson's‐related programmes rather than designed explicitly to support medication adherence.

The characteristics of included studies and interventions are summarised in Table 1. Intervention types and delivery modes are described below, with outcome measures and evidence relating to the potential of educational interventions to support medication adherence presented in subsequent sections.

**Table 1:** Characteristics of studies included in the scoping review.

Author	Country	Study design	Educational intervention focus	Delivery mode	Outcomes related to medication adherence
Connor et al., [30]	United States	Descriptive analysis of an RCT intervention	Care Coordination for Health Promotion and Activities in Parkinson's Disease (CHAPS)	Nurse‐led, telephone‐administered	Adherence inferred via care indicators
De Pandis et al., [31]	Italy	Randomised controlled trial	Therapeutic education embedded within a multidisciplinary rehabilitation programme	Face‐to‐face group education (People with Parkinson's and carers)	Adherence inferred via symptom control
Faurie et al., [32]	France	Mixed‐methods longitudinal study	Group therapeutic workshops with emphasis on medication	Face‐to‐face group workshops delivered by multidisciplinary Parkinson's specialists	Knowledge/beliefs related to adherence
Fincher et al., [33]	United States	Mixed methods	Standardised nurse‐led medication education	Nurse‐led via videophone	Acceptability and perceived usefulness of medication education
Grosset and Grosset, [34]	Scotland (UK)	Randomised controlled trial	Medication counselling and education focused on dose timing	MEMS pill bottles, verbal, and written education	Adherence measured directly (medication timing)
Lakshminarayana et al., [35]	UK	Randomised controlled trial	Smartphone‐based self‐management and medication support	Digital (smartphone application)	Self‐reported medication adherence.
Lindskov, Westergren and Hagell, [36]	Sweden	Non‐randomised controlled trial	Multidisciplinary education including medication‐related content	Face‐to‐face groups of 6–8 people with similar age, disease duration, and PD severity	No adherence‐related outcome
Sunvisson et al., [37]	Sweden	Non‐randomised experimental study	Outpatient educational programme incorporating psychosocial support	Group‐based, matched by PD severity	No adherence‐related outcome

The following results are presented in relation to the four research questions, combining descriptive mapping of intervention types and outcomes with an interpretive synthesis of adherence‐related findings and reported barriers and facilitators.

### 3.1 RQ1: What Different Types of Educational Interventions Have Been Developed to Support Medication Adherence for People With Parkinson's?

Educational interventions varied in scope, delivery mode, and focus. Four interventions focused primarily on Parkinson's medication education [32, 33, 34, 35], while the remainder incorporated medication‐related education within broader programmes addressing aspects of living with Parkinson's such as mobility, symptom management, coping strategies, daily living, and support systems [30, 31, 36, 37]. Medication education was commonly embedded within wider self‐management or rehabilitation programmes rather than delivered as a standalone intervention.

Interventions were delivered either through group‐based outpatient programmes (*n* = 4) or at‐home approaches (*n* = 4). Group‐based education was delivered face‐to‐face and typically facilitated by multidisciplinary Parkinson's specialists, including movement disorder specialists, nurses, and allied health professionals [31, 32, 36, 37], with one intervention tailored by disease stage [37]. At‐home interventions included nurse‐led telephone or videophone education [30, 33], electronic pill bottles combined with verbal and written education [34], and a smartphone‐based self‐management application [35].

Intervention duration and intensity varied considerably. Interventions focusing solely on medication education were typically delivered as single sessions, whereas broader programmes involved repeated contact over extended periods. Nurse‐led telephone and videophone interventions ranged from a brief consultation [33] to sustained support over many months [30].

Only one intervention was delivered digitally. This smartphone‐based intervention combined medication reminders with broader self‐management functions, including symptom tracking, educational content, and feedback tools [35]. Medication‐related educational content was described at a general level and sourced from Parkinson's UK and the Cure Parkinson's Trust, with limited detail on specific strategies such as timing, side‐effect management, or regimen complexity [35]. Descriptions of digital medication education were limited within the included literature.

Across studies, medication education commonly addressed understanding medication regimens, the rationale for medication use, dose timing, side effects, and self‐management strategies. However, few studies provided detailed descriptions of medication‐related educational content. Only one study explicitly described its medication education, consisting of brief written information explaining levodopa and the importance of regular medication timing [34]; other studies referred more broadly to therapeutic or belief‐focused education without specifying content [32].

### 3.2 RQ2: What Evidence Has Been Reported Regarding the Potential of Educational Interventions to Support Medication Adherence in People Living With Parkinson's?

Evidence regarding the potential of educational interventions to support medication adherence was reported across studies through direct adherence outcomes, proxy indicators, and changes in knowledge, beliefs, and self‐management behaviours. Although all interventions included Parkinson's medication‐related education, adherence was not consistently measured as an outcome. Three randomised controlled trials measured medication adherence directly [31, 34, 35], with adherence assessed as a primary outcome in two studies and a secondary outcome in one. One longitudinal study examined changes in medication‐related knowledge and beliefs [32], and two studies evaluated broader educational programmes without measuring adherence‐related outcomes [36, 37].

#### 3.2.1 Direct and Proxy Measures of Medication Adherence

Lakshminarayana et al. [35] reported a significant improvement in self‐reported medication adherence following use of a smartphone‐based self‐management application, measured using the Morisky Medication Adherence Scale (MMAS‐8). Participants in the intervention group demonstrated greater improvements in adherence than those receiving treatment as usual, alongside high engagement with the digital intervention. The authors attributed these effects to the simplicity of the app design, its integration within routine clinical care, and improved understanding of the condition and treatment.

In one study, active counselling combined with written medication information led to significant improvements in medication timing adherence [34]. Objective measurement using electronic pill bottles showed better timing adherence in the intervention group, particularly for medications with complex dosing schedules.

In another study, improvements in daily ‘off’ hours (where symptoms worsen between medication doses) were reported following a therapeutic education programme embedded within a multidisciplinary rehabilitation intervention [31]. While medication adherence was not the primary outcome, the intervention group demonstrated improvements in adherence measured using the MMAS‐8 alongside reductions in ‘off’ time. Participants also experienced improvements in motor and non‐motor symptoms and functional independence, whereas outcomes worsened in the control group.

#### 3.2.2 Knowledge and Beliefs Related to Medication Adherence

In one study, changes in knowledge, beliefs, and concerns about Parkinson's medication were examined following therapeutic education [32]. Significant improvements in medication knowledge and reductions in beliefs regarding the harmfulness of medication were observed, measured using a bespoke knowledge questionnaire and the Beliefs about Medicines Questionnaire. These changes were maintained at follow‐up and were not observed in the peer‐support control group. The authors suggested that modifying beliefs and concerns about medication supported participants to take greater ownership of their treatment and become more vigilant in medication management.

#### 3.2.3 Intermediate Outcomes Relevant to Medication Adherence

Several studies did not measure adherence outcomes directly but reported outcomes relevant to medication management. One study [33] reported that participants valued nurse‐led videophone or telephone education for improving understanding of medication regimens, side effects, and scheduling, supporting self‐management strategies. Similarly, another study [30] found that medication management was one of the most frequently requested topics within nurse‐led telephone support, with improved understanding supporting self‐care behaviours and communication with healthcare professionals.

#### 3.2.4 Reported Empowerment, Confidence, and Self‐Management Outcomes

Several interventions reported outcomes relating to empowerment [31], improved understanding and knowledge of medication [33, 36, 37], and enhanced confidence and self‐management behaviours [36, 37]. Several studies reported that participants valued education delivered by clinicians or perceived experts, and interventions embedded within routine care were viewed as more credible and acceptable [30, 31, 32, 33, 35].

### 3.3 RQ3: In Addition to Medication Adherence, What Other Outcomes Have Been Used to Assess the Effectiveness of Educational Interventions About Medication Adherence for People Living With Parkinson's?

Across the included studies, a wide range of outcomes were used to evaluate educational interventions. While three studies explicitly measured medication adherence [31, 34, 35], several others assessed adherence‐related constructs such as medication knowledge, beliefs, symptom control, or care indicators [30, 31, 32, 33]. Other studies focused on more general outcomes including participation, coping, and perceived benefit of education, without reporting outcomes relevant to medication adherence [36, 37].

#### 3.3.1 Quality of Life and Activities of Daily Living

Half of the included studies assessed quality of life or activities of daily living as outcomes. Quality of life was most commonly measured using the Parkinson's Disease Questionnaire (PDQ‐39) [31, 34, 35], while activities of daily living were assessed using the Sickness Impact Profile [37]. Findings were mixed: two studies reported significant improvements in quality of life or daily functioning following educational interventions [31, 37], one found no significant change [35] and another reported a deterioration in quality‐of‐life scores post‐intervention [34].

##### 3.3.1.1 Care and Consultation

Outcomes relating to care quality and clinical consultation were examined explicitly in two studies [30, 35] and discussed qualitatively in several others [31, 32, 33]. One study measured care quality through adherence to Parkinson's quality care indicators and reported improvements following nurse‐led education and coaching [30]. Another found that participants' perceptions of clinical consultations improved significantly following use of a smartphone‐based self‐management application, with participants reporting greater involvement in decision‐making and improved communication with clinicians [35].

##### 3.3.1.2 Symptoms and Mobility

Four studies measured changes in motor or non‐motor symptoms and mobility as outcomes [31, 34, 35, 37]. Only one study reported notable symptom improvements attributable to the intervention, showing sustained reductions in daily ‘off’ hours linked to improved medication adherence [31]. In contrast, the remaining studies reported no significant improvements in symptoms, despite evidence of improved adherence in some cases.

##### 3.3.1.3 Beliefs, Knowledge, and Psychosocial Outcomes

One study explicitly measured beliefs and knowledge about Parkinson's medication as outcomes [32], reporting significant improvements in medication knowledge and reductions in medication‐related concerns following therapeutic education. One study assessed psychosocial outcomes directly, reporting improvements in psychosocial situation following education focused on coping strategies and stress management [37]. These outcomes were less commonly measured across the literature.

### 3.4 RQ4: What Barriers and Facilitators for Successful Implementation of Educational Resources to Support Parkinson's Medication Adherence Can Be Identified?

To address RQ4, barriers and facilitators were inductively grouped into implementation‐related thematic categories, reflecting recurring patterns across the included studies. Identified factors related primarily to mode of delivery, convenience and accessibility, use of technology, and resource and staffing requirements. Although reporting was inconsistent across studies, common patterns emerged.

#### 3.4.1 Mode and Context of Delivery

A key implementation challenge related to where and how education was delivered. Some studies highlighted that delivering medication education during routine clinical appointments was suboptimal, due to time constraints, appointment‐related fatigue, and limited opportunity for person‐centred or empowering education [33, 35]. Another study highlighted the importance of tailoring education to individual learning needs and preferences, identifying this as a facilitator of self‐management when addressed, and a barrier when overlooked [30].

#### 3.4.2 Accessibility, Convenience, and Use of Technology

Educational interventions broadly followed one of two delivery approaches: face‐to‐face group workshops or at‐home interventions. Group‐based workshops required participants to travel and attend sessions. However, travel was not explicitly reported as a barrier in the included studies, and reasons for non‐participation were rarely described.

At‐home interventions aimed to reduce these barriers by eliminating the need for travel. In one study, participants described technology‐based education as more convenient than attending in‐person sessions [33]. However, technology also introduced new challenges: some participants experienced technical difficulties with videophone equipment [33], and digital interventions required access to and confidence in using compatible devices. Despite this, once initial technical challenges were resolved, technology‐based education was generally perceived as valuable and acceptable [35].

#### 3.4.3 Ongoing Staffing Requirements

The non‐digital interventions depended on ongoing input from trained healthcare professionals to deliver workshops, counselling, telephone support or care management. In contrast, the smartphone‐based intervention [35] enabled participants to access educational content and self‐management support without requiring direct professional delivery each time it was used, although clinicians supported its setup and incorporated participant‐generated data into follow‐up consultations.

## 4 Discussion

This discussion synthesises the findings of the scoping review in relation to wider literature on medication adherence, behaviour change, and intervention design. Rather than evaluating effectiveness, it focuses on identifying key patterns, gaps, and mechanisms to inform future phases of this research. Medication adherence is a multidimensional phenomenon influenced by patient, therapy, condition, health system, and socio‐economic factors [15], particularly in chronic conditions such as Parkinson's, where multimorbidity and polypharmacy complicate treatment regimens [6].

Although only a small number of studies measured medication adherence directly, the review identified a limited but emerging evidence base regarding the potential of educational interventions to support medication adherence in Parkinson's.

The Parkinson's‐specific literature identified here reflects challenges consistent with wider adherence research, notably the importance of education and the contextual fit of interventions. The reliance of non‐digital interventions on ongoing professional delivery may have implications for service capacity and scalability. Digital resources may enable repeated access to educational content without direct professional input each time they are used, while remaining integrated with appropriate clinical care.

### 4.1 Including Care Partners

The reviewed literature focused primarily on educating people living with Parkinson's, with care partners typically positioned in a supportive role rather than as direct recipients of education. This is notable given that informal carers are frequently involved in medication management for people with more advanced Parkinson's or are likely to assume this role as the condition progresses. This pattern reflects broader chronic illness literature recognising family members and informal carers as central to long‐term treatment management and adherence support [15].

Across the included studies, care partners did participate in some interventions, indicating willingness to engage with educational content. In one study, reductions in caregiver burden were reported when the person with Parkinson's participated in an educational intervention [31], suggesting that carers may also benefit indirectly. This aligns with wider evidence showing that involvement of informal carers in health education and self‐management interventions can support both patient outcomes and caregiver wellbeing, particularly where responsibility for treatment is shared or increases over time [38].

In the context of future digital interventions, it is plausible that family members may be increasingly able to access and engage with educational tools as Parkinson's progresses. These findings highlight the importance of recognising informal carers not only as supporters, but as key stakeholders in the design and delivery of educational interventions to support medication adherence in Parkinson's.

### 4.2 Involving People With Lived Experience of Parkinson's

While care partners may provide practical and functional support in medication management, people with lived experience can offer experiential, relational, and motivational support. Across the reviewed literature, only one study explicitly incorporated people living with Parkinson's into intervention delivery [32]. This study included ‘experts by experience’ who shared practical medication‐management strategies, demonstrating the value of experiential knowledge alongside clinical information. Participants reported valuing peer expertise, suggesting that lived experience was perceived as a meaningful and credible component of educational delivery.

The limited use of this approach across the remaining studies suggests that patient involvement in intervention delivery is not typically considered a core component of Parkinson's medication education. This contrasts with evidence from wider chronic illness research demonstrating the benefits of peer support for self‐management, empowerment, and confidence [39, 40]. Peer‐led or peer‐supported approaches have been shown to enhance self‐efficacy by normalising challenges, reducing stigma, and providing relatable examples of coping and adaptation [39].

In the context of Parkinson's, experiential input from peers may also help people reframe their relationships with medications, supporting more positive beliefs and reducing negative associations. The limited inclusion of lived experience within the reviewed interventions therefore represents a notable gap and highlights an opportunity to strengthen educational approaches by integrating peer expertise in ways that align with empowerment and self‐efficacy in long‐term condition management.

### 4.3 Absence of Co‐Design in Parkinson's Medication Education

None of the reviewed interventions reported collaboration with people living with Parkinson's or their informal carers during design or development. Co‐design is a collaborative process that involves stakeholders equally in intervention creation [41], and in the context of Parkinson's medication education, patients, carers, and healthcare professionals represent key stakeholders. However, the reviewed interventions were typically designed by healthcare professionals, with no evidence of patient or carer involvement in shaping content, format, or delivery.

Evidence from health and social care literature shows that involving people with lived experience in intervention design improves relevance, applicability, and outcomes [42, 43]. Designing interventions *with* rather than *for* people helps ensure alignment with the needs of the target population [44]. The absence of co‐design across the reviewed studies therefore represents a significant gap in the literature and highlights participatory approaches as an important area for future development in supporting medication adherence in Parkinson's.

### 4.4 Medication Adherence as a Behavioural and Contextual Challenge: Interpreting the Findings Through a Self‐Efficacy Lens

Medication adherence in Parkinson's is particularly challenging due to the need for frequent, time‐sensitive dosing. Adherence is widely recognised as a behavioural process influenced by motivation, beliefs, and confidence, rather than knowledge alone [15]. Educational approaches focused solely on information provision are unlikely to support sustained behaviour change. In contrast, interventions informed by behaviour change are more likely to address health behaviour and demonstrate lasting effects [45, 46], with systematic reviews showing greater improvements in medication adherence when theory or behaviour change techniques are incorporated [47, 48].

A key gap identified across the studies was the absence of explicit use of behaviour change theory in the design of the interventions. Although several interventions aimed to empower participants by supporting self‐management and coping strategies, none articulated a formal theoretical framework (behavioural or otherwise) to guide intervention development.

Given the complexity of medication adherence in Parkinson's and the limits of education alone, concepts such as empowerment and self‐efficacy may provide useful frameworks for interpreting the findings of this review. Although self‐efficacy was not directly measured in any included study, several interventions reported outcomes relating to empowerment, confidence, knowledge, communication, and self‐management behaviours. These outcomes are conceptually aligned with constructs commonly associated with self‐efficacy [49] and informed consideration of self‐efficacy within the wider research programme.

Self‐efficacy, defined as an individual's belief in their capacity to perform behaviours required to achieve desired outcomes [49], is a well‐established determinant of health behaviour [50] and is particularly relevant in Parkinson's, where people have been shown to experience lower levels of self‐efficacy than their peers [51]. Across the included studies, educational interventions frequently reported outcomes relating to empowerment, knowledge, confidence, and self‐management [31, 32, 33, 36, 37]. Within the wider literature, empowerment has been described as a process that can support the development of self‐efficacy through increased knowledge, competence, and perceived control. Educational approaches that emphasise empowerment have been shown to promote personal change, self‐determination, competence, and a sense of mastery in people living with long‐term conditions [52].

For people living with Parkinson's, understanding their condition, prescribed medications, and potential side effects may support greater confidence in medication management and self‐management behaviours. Educational interventions may also contribute to improved communication with healthcare professionals and greater involvement in treatment decisions. By improving understanding and reducing negative perceptions, educational interventions may contribute to the development of a therapeutic alliance, where individuals feel empowered to manage their treatments rather than adhering to prescribed regimens out of obligation. This empowerment may improve the quality of clinical consultations, enhancing communication between people with Parkinson's and caregivers and supporting better medication management. Across studies, education appeared to support more effective interactions between people with Parkinson's and healthcare professionals, contributing to perceived improvements in care. These findings align with the WHO's identification of patient‐provider communication and patient education as key determinants of medication adherence [15].

Behaviour change theory has been successfully applied to educational interventions targeting medication adherence across a range of long‐term conditions, including cardiovascular disease, diabetes, chronic pain, stroke, and paediatric conditions [53, 54, 55]. Drawing on this broader evidence base, future educational interventions to support Parkinson's medication adherence may be strengthened by explicitly incorporating behaviour change theory to enhance self‐efficacy in both people with Parkinson's and their caregivers.

**Figure 2:**
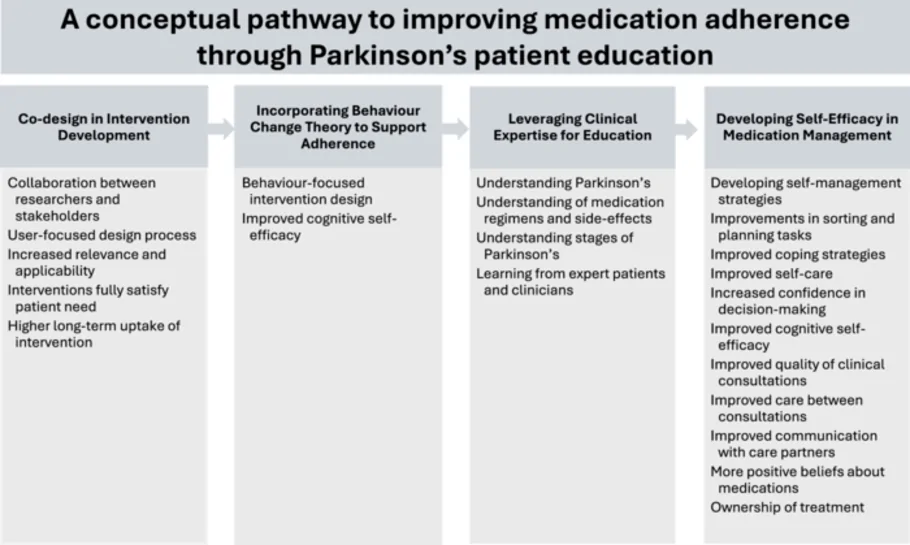
A conceptual pathway to improving medication adherence through Parkinson's patient education. Proposed conceptual pathway developed through integrative synthesis of the review findings.

Figure 2 presents a proposed conceptual pathway for improving medication adherence through Parkinson's patient education, developed through an integrative synthesis of the review findings. While the included interventions were heterogeneous and rarely theory‐driven, recurring patterns across studies enabled the identification of common mechanisms and gaps that informed the structure of this pathway.

### 4.5 Summary and Integration of Findings

This scoping review suggests that educational interventions for Parkinson's medication adherence operate through interrelated cognitive, affective, behavioural, and relational processes, rather than through knowledge provision alone. Although the reviewed interventions were heterogeneous and did not report the explicit use of formal theoretical frameworks, recurring patterns indicate that changes in self‐management, confidence, beliefs about medications, and communication are plausibly linked to enhanced self‐efficacy. Viewed collectively, these patterns support a conceptual interpretation of how educational intervention characteristics may indirectly influence medication adherence, despite persistent gaps in theory use, co‐design, and outcome consistency. As a scoping review, this work does not assess intervention effectiveness or quality but instead maps the nature and mechanisms of existing educational approaches. This evidence provides a foundation for subsequent co‐design and theory‐informed development of educational interventions to support medication adherence in Parkinson's. These findings should be understood within the context of broader, non‐evaluated supports that people with Parkinson's may access in everyday life, which were beyond the scope of this review.

## 5 Strengths and Limitations

This scoping review addresses an internationally recognised priority to improve medication adherence and access to treatment for people with neurological conditions, including Parkinson's, as highlighted by the WHO [15] and provides implementation‐relevant insights through comprehensive mapping of evidence. However, the limited and heterogeneous nature of the evidence base, with few interventions directly measuring adherence, constrains conclusions about effectiveness and highlights gaps in current provision. A further limitation of the evidence base was the substantial variability in outcome selection and measurement. Quality of life, symptoms, care‐related outcomes, medication knowledge, and adherence were assessed inconsistently across studies, limiting comparability and making it difficult to determine which outcomes are most responsive to educational interventions. Future research would benefit from greater alignment between intervention aims, outcomes, and proposed mechanisms of change. Additionally, title and abstract screening was conducted by one reviewer, which may have increased the risk of selection bias or inadvertent exclusion of eligible studies; however, this risk was mitigated through team development of the search strategy and eligibility criteria and second‐reviewer verification of full‐text decisions. Although an a priori protocol was developed to guide the review, it was not prospectively registered or published, which limits transparency and the ability to independently verify adherence to the planned methods.

## 6 Conclusion

This scoping review demonstrates that medication adherence in Parkinson's is a complex and multifactorial behaviour, shaped by interacting clinical, behavioural, social, and contextual influences. Across the reviewed literature, educational interventions that combined credible clinical input with empowering approaches were associated with positive outcomes, suggesting that supporting people with Parkinson's to develop understanding, confidence, and a sense of control over their medication regimens is an important component of adherence support. Within this complexity, self‐efficacy emerged as a potentially useful theoretical framework through which the relationship between education and medication‐taking behaviour may be understood.

However, the review also highlights important gaps in the existing evidence base. Educational interventions were heterogeneous, rarely theory‐informed, and did not report the use of co‐design approaches involving people living with Parkinson's or their informal carers. Outcome measurement was also inconsistent, with limited direct assessment of medication adherence and related behavioural mechanisms. These limitations constrain the ability to draw conclusions about effectiveness and underline the need for more systematically developed interventions.

A further challenge concerns the delivery of educational support in ways that are accessible and acceptable to people living with Parkinson's. While group‐based workshops may facilitate engagement for some individuals, they may also introduce barriers related to travel, fatigue, and service capacity. In contrast, educational resources that can be accessed flexibly and used at home may help to reduce these barriers, particularly when they are positioned as an extension of clinical care rather than a replacement for it. This highlights the potential value of digital educational approaches that are grounded in clinical expertise and responsive to everyday lived experience.

These findings suggest that while no single intervention is likely to address the full complexity of medication adherence in Parkinson's, self‐efficacy represents a theoretically grounded and meaningful construct through which the impact of educational interventions can be understood. Focusing on self‐efficacy in future intervention development offers a way to link education, behaviour change, and patient experience, while remaining attentive to the broader social and contextual factors that shape medication management.

It is also important to acknowledge that medication non‐adherence may arise from external and systemic factors, such as challenges in medication delivery or administration within healthcare settings, which lie beyond the scope of patient‐focused educational interventions and are being addressed through wider service‐level and professional education initiatives.

This review aligns with international priorities to strengthen medication management and self‐management support for neurological conditions, including Parkinson's [15] and provides a foundation for the subsequent co‐design and theory‐informed development of educational resources to support medication adherence in Parkinson's.

## Author Contributions

**Hazel Haworth:** conceptualization, investigation, writing – original draft, methodology, formal analysis, writing – review and editing. **Gillian Carter:** conceptualization, funding acquisition, supervision, methodology, writing – review and editing. **Katherine M. A. Rogers:** writing – review and editing, supervision. **Gary Mitchell:** writing – review and editing. **Laura Jacobs:** writing – review and editing. **Patrick Stark:** conceptualization, methodology, supervision, validation, writing – review and editing, funding acquisition.

## Consent

Patient consent was not required as this study involved secondary analysis of published literature.

## Ethics Statement

The authors have nothing to report.

## Conflicts of Interest

The authors declare no conflicts of interest.

## Patient and Public Involvement

Patients and/or the public were not involved in the conduct of this scoping review. However, people living with Parkinson's and their carers have been integral to the wider study within which this review is situated.

## Supporting information


Supporting File 1

Supporting File 2

## Data Availability

The data that support the findings of this study are available in the supporting material of this article.
