# Supplementary material for: Supporting Medication Adherence in Parkinson's Through Education: A Scoping Review of Interventions
Source: Health Expect. 2026 Aug 30;29(5):e70852. doi: 10.1111/hex.70852 (PMC13527395; doi:10.1111/hex.70852)
Supplement: Supplementary file 2 [file HEX-29-e70852-s002.docx]

| Supplementary Table S2 |  |
| --- | --- |
| Search Strategy |  |
| Key search areas | MeSH Terms |
| Parkinson’s Disease | Parkinson* |
|  | AND |
| Medication management | Medication compliance OR  Medication adherence OR  Medicine OR  Medication management OR  Medication regimen OR  Medication routine OR  Medication non-adherence OR  Medication non-compliance OR  Prescription management OR  Drug adherence OR  Drug compliance OR  Treatment compliance OR  Treatment adherence OR  Medication |
|  | AND |
| Education | Education OR  Patient education OR  Health education OR  Health promotion OR  Health literacy OR  Health knowledge OR  Health information OR  Digital education OR  Assistive technology OR  Internet-Based intervention OR  Web-based intervention OR  World wide web applications OR  Telehealth OR  Telemedicine OR  Mobile applications OR  Smartphone OR  Cellular phone OR  Online education OR  Online services |
